# Three-Dimensional Environment Mapping with a Rotary-Driven Lidar in Real Time

**DOI:** 10.3390/s25154870

**Published:** 2025-08-07

**Authors:** Baixin Tong, Fangdi Jiang, Bo Lu, Zhiqiang Gu, Yan Li, Shifeng Wang

**Affiliations:** 1 School of Optoelectronic Engineering, Changchun University of Science and Technology, Changchun 130022, China; 2023100364@mails.cust.edu.cn (B.T.); jiangfangdi@outlook.com (F.J.); 2024100410@mails.cust.edu.cn (Z.G.); 2 Zhongshan Institute of Changchun University of Science and Technology, Zhongshan 528400, China; barrylu2015@outlook.com; 3 Faculty of Computing, Macquarie University, Sydney 2109, Australia; y.li@mq.edu.au

**Keywords:** simultaneous localization and mapping, multi-sensor fusion, point cloud, loop closure detection

## Abstract

Three-dimensional environment reconstruction refers to the creation of mathematical models of three-dimensional objects suitable for computer representation and processing. This paper proposes a novel 3D environment reconstruction approach that addresses the field-of-view limitations commonly faced by LiDAR-based systems. A rotary-driven LiDAR mechanism is designed to enable uniform and seamless full-field-of-view scanning, thereby overcoming blind spots in traditional setups. To complement the hardware, a multi-sensor fusion framework—LV-SLAM (LiDAR-Visual Simultaneous Localization and Mapping)—is introduced. The framework consists of two key modules: multi-threaded feature registration and a two-phase loop closure detection mechanism, both designed to enhance the system’s accuracy and robustness. Extensive experiments on the KITTI benchmark demonstrate that LV-SLAM outperforms state-of-the-art methods including LOAM, LeGO-LOAM, and FAST-LIO2. Our method reduces the average absolute trajectory error (ATE) from 6.90 m (LOAM) to 2.48 m, and achieves lower relative pose error (RPE), indicating improved global consistency and reduced drift. We further validate the system in real-world indoor and outdoor environments. Compared with fixed-angle scans, the rotary LiDAR mechanism produces more complete reconstructions with fewer occlusions. Geometric accuracy evaluation shows that the root mean square error between reconstructed and actual building dimensions remains below 5 cm. The proposed system offers a robust and accurate solution for high-fidelity 3D reconstruction, particularly suitable for GNSS-denied and structurally complex environments.

## 1. Introduction

Three-dimensional reconstruction [1] refers to the creation of mathematical models of three-dimensional objects suitable for computer representation and processing. It is also a key technology for building virtual reality that represents the objective world in a computer. In computer vision, laser-based 3D imaging involves measuring the distance to surfaces in the environment by emitting laser pulses and capturing the time-of-flight for each reflected signal. A single-point laser rangefinder acquires distance data point by point, and by combining this with the known orientation of the sensor during scanning, the spatial position of each point can be reconstructed. When the laser is mechanically swept or mounted on a rotating platform, a full 3D point cloud can be obtained over time. Simultaneous Localization and Mapping (SLAM) [2] plays a crucial role in laser-based 3D reconstruction, as it enables robots to localize themselves in unknown environments in real time while simultaneously building detailed and accurate 3D maps. Especially when combined with LiDAR (Light detection and ranging) technology, the robot is able to perform high-precision measurements using actively emitted pulsed lasers. It maintains excellent stability and accuracy even when external lighting conditions change significantly. From home service robots to industrial automation systems to self driving cars, 3D reconstruction technology provides the necessary spatial awareness for these applications.

SLAM technology has achieved remarkable results in the field of 3D reconstruction. As the LOAM series of methods are widely used, they typically place the LiDAR horizontally on a wheeled mobile platform. However, the limitation of the field of view (FOV) [3] of LiDAR has become an urgent problem. Since LiDAR has a limited vertical or horizontal field of view, it is unable to capture objects or environmental features outside its field of view [4,5]. This results in a reconstructed 3D model that may be incomplete and missing important details. In addition to the field-of-view limitations [6], the cumulative error of the sensor during use becomes a key factor that affects the accuracy of 3D reconstruction. To reduce the cumulative error, researchers have proposed the method of loop closure detection. Loop closure detection is designed to help robots recognize scenes that have been reached before, enabling closed-loop correction of the map and thus eliminating cumulative errors. For LiDAR loop closure detection, the most commonly used method is still Scan Context [7]. But with Scan Context, there are some disadvantages, i.e., it only utilizes the maximum height, lacking point cloud information. It also does not have rotational invariance and cannot effectively handle point cloud data with large rotations. In this case, the matching result may be affected by the rotation, leading to an increase in the matching error and affecting the localization accuracy of the SLAM system.

**Figure 1:**
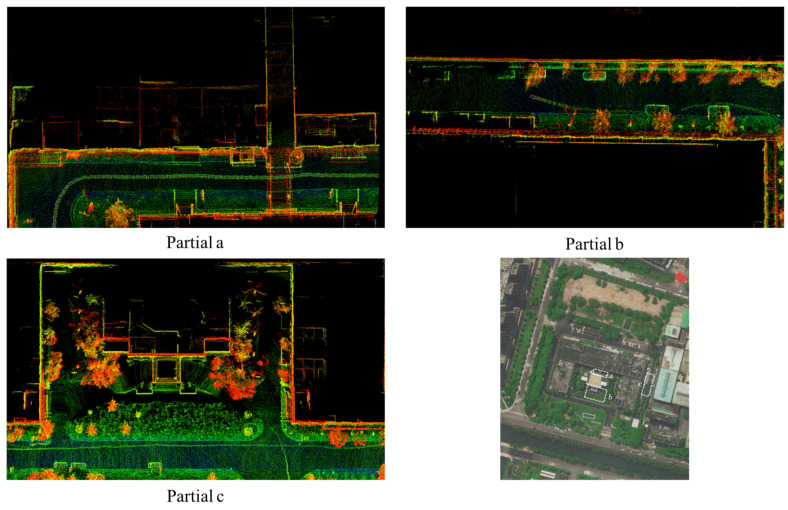
Real-time mapping results of our system in an outdoor environment, which is about 1.5 km in length and contains various dynamic obstacles. Three partial LIDAR maps show visually consistent maps due to the full-range scanning and accurate loop closures.

To overcome the problems in the existing technology, an innovative rotary-driven LiDAR system is designed. The system is based on slip ring technology. The LiDAR is rotated by a stepper motor to acquire uniformly distributed full-field-of-view point cloud data. To enhance the registration accuracy across consecutive frames, we propose a multi-threaded feature registration method tailored for the rotary-driven LiDAR system. Specifically, each LiDAR scan is divided into 12 equal angular segments, and within each segment, feature extraction is performed to identify both edge and planar points. This strategy addresses the degradation in registration accuracy caused by uneven spatial distribution of features in the point cloud, ensuring a more consistent and robust feature representation across the entire field of view. Uneven feature distribution refers to the fact that in the point cloud, some regions have dense feature points while other regions lack feature points, which makes feature matching and registration difficult. In order to further improve the loop closure detection accuracy and effectively handle point cloud data with a large rotational amplitude, a two-staged loop-closure algorithm is used to reduce the cumulative error of the system. In the first stage, the system utilizes the visual BoW2 (Bag of Words) [8,9] model to perform preliminary feature matching and quickly narrow down the matching range. In the second stage, the system combines feature extraction from LiDAR to perform more accurate feature matching, thus realizing high-precision position estimation and loop closure detection. Figure 1 shows the point cloud map constructed in real time, demonstrating that the LV-SLAM integrated with the rotary-driven LiDAR system can generate a highly consistent map of the complex scene under the long operation. The main contributions of this work are as follows:

(1) A novel rotary-driven LiDAR system is developed, which utilizes a slip-ring mechanism for continuous rotation and thus generating uniform point clouds.
(2) Multi-threaded feature registration and a two-stage closed-loop algorithm based on multi-sensor fusion are proposed.
(3) The developed system and algorithms have been extensively tested using the public KITTI dataset and our custom-made mobile platform, meaning that the 3D reconstruction results are highly visually consistent. The system has higher robustness and accuracy.

## 2. Related Works

Scanning 3D imaging LiDAR is composed of a single-point laser ranging system with a beam scanning device, which is a relatively mature 3D imaging LiDAR technology. Several LiDAR systems [10,11] have been proposed by researchers in order to obtain complete three-dimensional environmental information. Kaul [12] has developed a general-purpose airborne 3D reconstruction system for small quadcopters. The system utilizes an innovative passive drive mechanism and continuous-time SLAM technology to generate accurate, dense point cloud maps without GPS/INS. Its reliance on the quadcopter’s rotor down suction for rotation limits scanning speed and resolution, affecting the efficiency and quality of data acquisition. In addition, the data cannot be processed in real time. Bosse [13] has designed a reconstruction system called Zebedee. It extends the field of view beyond the standard scanning plane by coupling a 2D laser ranging scanner with an inertial measurement unit and mounting it on a spring, utilizing body acceleration and vibration. This provides a mechanically simple, lightweight and relatively low-cost 3D perception solution for robots. The Zebedee system relies on body acceleration and vibration to propel the 2D scanner, which can be affected by external disturbances and instabilities, resulting in reduced data accuracy and stability. Karimi [14] proposed a low-latency LiDAR SLAM framework called LoLa-SLAM, which utilizes sliced point cloud data from rotating LiDAR and a multi-threaded matching pipeline to achieve high update rate and low-latency 6D attitude estimation. Feature points are extracted by an innovative 2D Lissajous rotation pattern and 2D roughness model, and an attitude estimator combining a temporal motion predictor and an extended Kalman filter, which effectively improves the autonomous navigation performance of indoor UAVs. A 2D Lissajous rotational model is used to overcome the problem of limited field of view of LiDAR. However, this method cannot completely eliminate the blind zones and the rotational mode itself introduces specific dynamic errors.

Zhen [15] performs 3D reconstruction using a rotating multibeam lidar and combines it with a stereo camera for dense 3D reconstruction. The system has a very high resolution but needs to be stationary while collecting data. Li Yan [16] designs a rotary-driven LiDAR mapping system for UAV and backpack platforms. The improved closed-loop detection algorithm is based on the Cartographer’s algorithm, whose effectiveness depends on specific features of the environment and performs poorly in environments lacking distinctive features. For this reason, this paper designs a rotation-driven LiDAR system for wheeled mobile and handheld platforms, which can acquire uniform full-field-of-view point cloud data in real time. And the LiDAR motion compensation is performed by uniform rotation modeling with IMU pre-integration.

The data processing and reconstruction thereof are equally crucial, except for the mechanical structure design of the full-field-of-view (FOV) data acquisition system. Numerous LiDAR-based SLAM algorithms have been proposed to enhance the accuracy of 3D reconstruction. LOAM [17] is an efficient LiDAR odometry and map building method that runs in real time and generates highly accurate maps. It improves the accuracy of point cloud alignment by distinguishing between edge and planar features and processing them separately. LOAM is sensitive to objects in dynamic environments, which can lead to erroneous data correlation. LeGO-LOAM [18] is an improved version of LOAM designed for use on ground-based robots. It adds closed-loop detection to improve long-term localization accuracy. And the distinction between ground and planar features helps to filter out ground points in building maps. Similar to LOAM, LeGO-LOAM may encounter problems in dynamic environments and the high computational cost of closed-loop detection may affect real-time performance. LIO-SAM [19] is a tightly coupled LiDAR Inertial Measurement Unit (IMU) SLAM algorithm that utilizes IMU data to supplement LiDAR data for improved robustness and accuracy in dynamic environments. The performance of LIO-SAM is highly dependent on the quality and calibration of the IMU. LVI-SAM [20] integrates LiDAR, IMU, and vision camera data, which improves the robustness and accuracy of SLAM systems through multi-sensor fusion, especially in environments rich in visual features. LVI-SAM requires precise time synchronization and sensor calibration. The main difference between this method and ours is the difference in the processing of the acquired data, and its inability to be adapted to rotationally driven lidar systems. NERF-LOAM [21] is an algorithm that combines LOAM and Neural Radiation Field (NeRF), which utilizes an implicit representation of NeRF to optimize the alignment and reconstruction of point clouds, improving the visualization and accuracy of maps. The training and inference process of NERF-LOAM is computationally costly and requires a large amount of data to train the NeRF model, which makes it unsuitable for real-time applications or resource-limited platforms. Recent works also explore 3D deep learning-based reconstruction in human–machine interaction scenarios [22], demonstrating the effectiveness of combining semantic features with point cloud geometry. In rehabilitation fields, 3D deep networks have also shown promise in real-time applications under complex conditions. The current mainstream LOAM series algorithms are not adapted to rotating LiDAR data except for LOAM, which has no rotating LiDAR dataset.

## 3. Methodology

**Figure 2:**
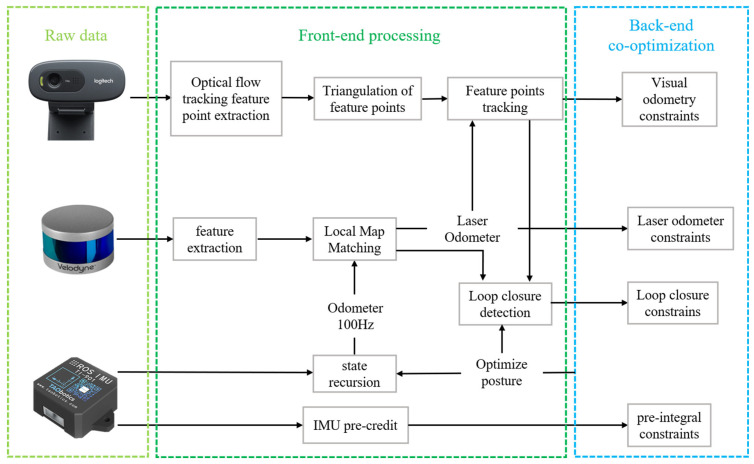
SLAM processing pipelines showing the LiDAR, camera, and IMU data processing, front end, and back-end processing.

In this section, we present the methodology used in the multi-sensor fusion SLAM system, which is specifically tailored for rotary-driven LiDAR systems. The system architecture is shown in Figure 2. The proposed rotary-driven LiDAR system is built upon a slip-ring mechanism that enables uninterrupted 360° rotation. The mechanical structure consists of a rigid platform mounted with a Velodyne VLP-16, driven by a NEMA-17 stepper motor. The choice of a stepper motor is based on its precise incremental rotation and low-cost control. The motor is controlled via a micro-stepping driver with a closed-loop PID controller to ensure smooth acceleration and deceleration. To ensure uniform and stable scanning, a trapezoidal velocity profile is adopted, and real-time motor position feedback is used for adjusting rotation speed dynamically based on scan completion timestamps. The slip ring ensures uninterrupted data and power transmission during continuous motion. Through careful alignment and vibration isolation pads, the system minimizes motion artifacts in the collected data.

The LV-SLAM system employs a modular, multi-threaded architecture, where each sensor stream—LiDAR, IMU, and Camera—is independently handled and time-synchronized using a shared ROS timebase. The feature registration thread employs a synchronized buffer queue, where point cloud frames are processed using curvature-based segmentation and associated with visual features extracted via ORB descriptors. Each thread communicates through a shared memory buffer protected by mutexes to avoid race conditions. For loop closure, the first stage uses a BoW2 index trained on ORB descriptors to rapidly detect candidate frames. The second stage computes ICP alignment between LiDAR features for refined closure. A feature similarity threshold of 0.35 (empirically tuned) is set for BoW2 matches, and loop closures are triggered only if visual and LiDAR matching both exceed similarity thresholds. This ensures robustness against false positives.

### 3.1. Feature Extraction

During the acquisition of raw point cloud data, sensor motion may introduce abnormal values or noise, which can negatively impact the estimation of local features and lead to registration failure. Therefore, it is essential to remove outliers prior to feature extraction. In this work, we treat outliers as points whose local neighborhood distances deviate significantly from the global distribution. For each point $p_{i}$ in the point cloud set $S=\left\{p_{1},p_{2},\ldots ,p_{n}\right\}$, to determine the average distance between a point and its closest K neighbors, we hypothesize that the resulting distribution adheres to a Gaussian pattern, characterized by a mean μ and a standard deviation σ. Points with mean distances falling outside the range defined by the global mean distance plus or minus a factor of the standard deviation (μ−σ⋅std,μ+σ⋅std) are deemed outliers and excluded from the dataset. The formula for this computation is provided below:

(1) $$\mu =\frac{1}{n}\sum_{i=1}^{n}S_{i}$$

(2) $$\sigma =\sqrt{\frac{1}{n}\sum_{i=1}^{n}{\left(S_{i}-\mu \right)}^{2}}$$

where $S_{i}$ represents the distance between the $n_{th}$ point and any other point, K is the number of nearest neighbors and std represents a predefined parameter for standard deviation.

Our feature extraction approach integrates both image and point cloud data. (1) For image data, we utilize optical flow tracking to capture motion information between pixels, enabling accurate identification of corner points. These corner points often correspond to edges or prominent features of objects and serve as vital reference points for subsequent feature matching and tracking. (2) For the point cloud data, we differentiate between various types of points based on curvature information. Curvature is a significant metric for quantifying the degree of bending on an object’s surface. By computing curvature values for each point in the point cloud, we categorize them into corner points and surface points. Corner points exhibit higher curvature values, while surface points have relatively lower curvature values. The calculation formula is represented by Equation (3).

(3) $$c=\frac{1}{|S|\cdot X_{\left(k,i\right)}^{L}}\underset{j\in S,j\neq i}{\sum }X_{\left(k,i\right)}^{L}-X_{\left(k,j\right)}^{L}$$

where S is consecutive points obtained after outlier removal and down sampling of LiDAR data within the same frame. $X_{\left(k,i\right)}^{L}$ represents the i point in the point cloud $P_{k}$ scanned at the k iteration in the L coordinate system.

**Figure 3:**
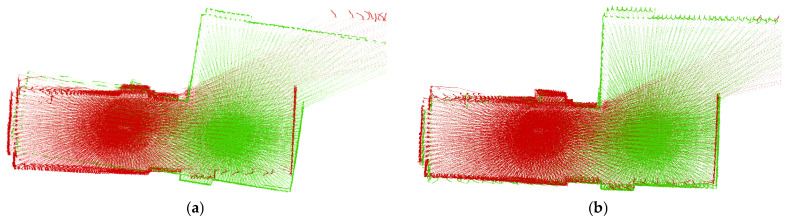
Registered point clouds with different methods: (**a**) traditional feature extraction and (**b**) our method. The red and green point clouds are two consecutive frames of a point cloud. Compared to traditional methods, we are able to register more accurately.

The paper introduces a multi-threaded feature extraction algorithm to accommodate the rotary-driven LiDAR system. This algorithm evenly partitions each LiDAR scan into twelve sections and selects four edge points and four plane points from each segment. This ensures that numerous feature points are extracted from all scanned regions, ultimately enhancing the precision of point cloud registration. Our method is compared with traditional algorithms as shown in Figure 3.

### 3.2. Motion Distortion Compensation

Point cloud distortion arises due to the carrier’s movement during LiDAR data acquisition, leading to points in the cloud being captured at different times. This results in inconsistencies in the coordinate systems of various laser points within the same frame. The motion of a rotary-driven LiDAR system can be broken down into two components: rotation and translation. To address this, we employ a uniform rotation model to correct rotational distortion and utilize IMU-assisted compensation to mitigate translational distortion.

#### 3.2.1. Rotational Distortion Compensation

Assuming a uniform velocity model, the coordinates of each point within the same frame of the point cloud can be determined using the rotation speed ω and the time t. The equation below represents the rotational speed of the stepper motor and t is an integral part of the information provided by the LIDAR point cloud. Using the coordinate information of each LIDAR point cloud, all point clouds can be transformed into the coordinate system corresponding to the initial moment of the frame through Equation (4).

(4) $$L_{s}=L_{p}\times T$$

where $L_{s}$ is the frame-initial coordinate system, $L_{p}$ is the coordinate of each point and T is the transformation matrix.

#### 3.2.2. Translational Distortion Compensation

The timestamp synchronization between LIDAR and IMU is performed first, and then each frame of IMU data corresponds to the displacement and velocity in the world coordinate system.

The IMU provides measured acceleration $a_{m}^{b}$ and angular velocity $\omega_{m}^{b}$ expressed in its body coordinate system with noise:

(5) $$\left\{\begin{array}{l} a_{m}^{b}=R_{w}^{b}\left(a_{t}^{w}-g^{w}\right)+b_{\alpha_{t}}+n_{\alpha }\\ \omega_{m}^{b}=\omega_{t}^{b}+b_{g_{t}}+n_{g} \end{array}\right.$$

where $R_{w}^{b}$ denotes the transformation matrix from the w and b systems; $a_{t}^{w}$ and $\omega_{t}^{b}$ are the true values of acceleration and angular velocity, respectively; $n_{a}$ and $n_{g}$ are the accelerometer and gyroscope measurement noise models as Gaussian white noise, with $n_{a}\sim N\left(0|,|\sigma_{a}^{2}\right),n_{g}\sim N\left(0|,|\sigma_{g}^{2}\right)$; $b_{a_{t}}$ and $b_{g_{t}}$ are the accelerometer and gyroscope bias models as a random walk process, respectively; and $g^{w}$ is the gravitational vector expressed in the world coordinate system. The IMU data between the adjacent frames are pre-integrated to reduce the computational complexity. The integration terms are pre-integrated as follows:

(6) $$\left\{\begin{array}{l} a_{b_{k+1}}^{b_{k}}=\iint_{t\in [\vec{k},\vec{k}+1]}R_{b_{t}}^{b_{t}}\left(a_{m}^{b}-b_{a_{t}}\right)dt^{2}\\ \beta_{b_{k+1}}^{b_{k}}=\int_{t\in [\vec{k},\vec{k}+1]}R_{b_{t}}^{b_{k}}\left(a_{m}^{b}-b_{a_{t}}\right)dt\\ \gamma_{b_{k+1}}^{b_{k}}=\int_{t\in [\vec{k},\vec{k}+1]}q_{b_{t}}^{b_{k}}\left(\omega_{m}^{b}-b_{E_{t}}\right)dt \end{array}\right.$$

where $a_{b_{\hat{k}+1}}^{b_{\hat{k}}}$, $\beta_{b_{\tilde{\kappa }+1}}^{b_{\tilde{\kappa }}}$ and $\gamma_{b_{\tilde{\kappa }+1}}^{b_{k}}$ are the pre-integrated position, velocity, and attitude, respectively.

Finally, the obtained motion information is used for interpolation, and the compensation transformation matrix of each laser point with respect to the laser point at the scanning moment of the starting point is calculated by Equation (4).

The rotary-driven LiDAR module provides denser, full-angle point clouds compared to fixed-mounted LiDAR. This increased data richness enhances the initialization and tracking performance of LV-SLAM. The continuous rotation ensures that key structural details are not occluded, allowing the SLAM system to maintain loop closure even in corridor-like or partially symmetric environments. Furthermore, rotational uniformity ensures temporally consistent input to the multi-threaded SLAM backend, reducing registration ambiguity. Thus, the hardware and software components are tightly integrated for mutual reinforcement: improved scans enable better loop closure, which in turn optimizes trajectory correction for the next scans.

### 3.3. Multi-Sensor Contact Optimization

The back-end module receives the front-end data and performs the optimization to enhance the accuracy. We jointly optimize the LiDAR odometry results as inter-frame constraints on top of the visual inertial odometry, instead of independently optimizing the visual inertial odometry and the laser inertial odometry, which enhances the computational performance of the system.

**Figure 4:**
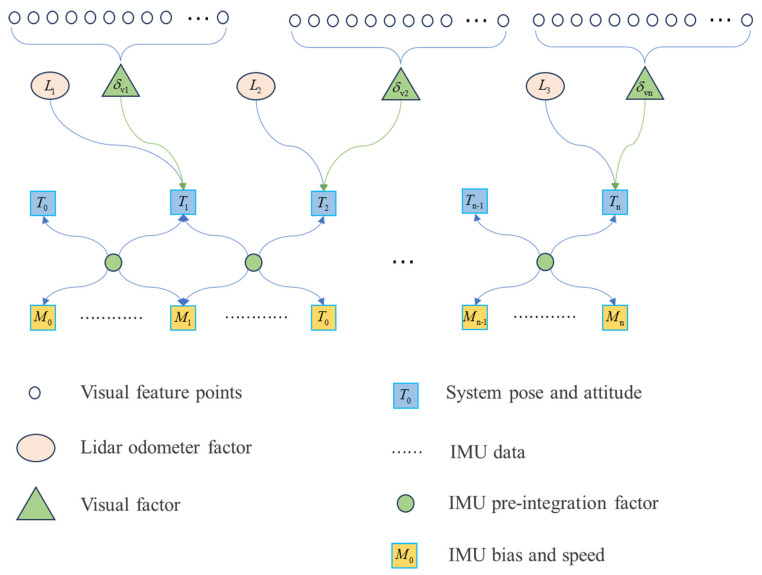
Structure of factor graph.

The back-end state estimation module employs a tightly coupled sliding window optimization algorithm to jointly optimize the observation constraints from the IMU, LiDAR, and vision. The state quantities maintained in the back-end are as follows:

(7) $$\left\{\begin{array}{ll} X= & \left[x_{b_{0}}^{w}x_{b_{1}}^{w}\ldots x_{b_{n}}^{w},\lambda_{0}\lambda_{1}\ldots \lambda_{m}\right]\\ x_{b_{k}}^{w}= & \left[p_{b_{k}}^{w}v_{b_{k}}^{w}q_{b_{k}}^{w}b_{a}b_{g}\right],k\in [0,n] \end{array}\right.$$

where $x_{b_{n}}^{w}$ denotes the k state vector of the IMU in the sliding window, which contains the position, velocity, and attitude represented in the world coordinate system; $b_{a}$ and $b_{g}$ are the accelerometer and gyroscope bias error of the IMU, respectively. The back-end also optimizes the inverse depth information of the visual feature point with m being the total number of visual feature points to be optimized in the sliding window. Figure 4 shows the constraints constructed by sensor observations in the back-end state estimation model. The sensor observations provide three categories of constraint factors, namely IMU pre-integration factor, visual reprojection constraint factor, and LiDAR odometry factor. They constrain the state quantities to be optimized in the sliding window, i.e., velocity, position, attitude, and IMU bias, respectively.

### 3.4. Two-Staged Loop-Closure Detection

The system uses a closed-loop detection method based on DBoW2 bag-of-words to recognize the location. To improve the performance of the system algorithm, the similarity between image frames is first compared. If the similarity score is greater than a threshold, the frame is determined to be a closed-loop frame. And the pose information of the current frame and the visual closed-loop candidate frame is sent to the LiDAR odometer as the initial value for closed-loop optimization. And the loop descriptor is extracted by laser features and the similarity score between two frame descriptors is calculated. Further confirm whether it is a loop frame or not.

**Figure 5:**
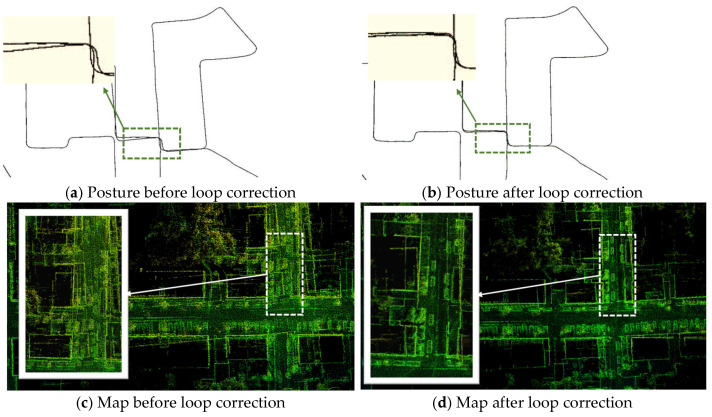
Comparison chart before and after loop correction. (**a**,**b**) clearly demonstrate a reduction in trajectory error upon looping back. (**c**,**d**) illustrate how looping back effectively resolves issues related to map offset and ghosting.

When mapping extensive environments, noise accumulation gradually increases errors. Figure 5a exhibits a trajectory without loop constraints, revealing poor overlap in the loop area, indicated by green lines, and a subpar fitting effect. Conversely, Figure 5b incorporates a loop detection module, correcting the initial offset and enhancing the fitting quality. Without loop constraints, Figure 5c demonstrates a noticeable “ghost” issue in the loop area, compromising the drawing quality. However, Figure 5d integrates the loop detection module, effectively resolving the ghost problem and enhancing the drawing’s clarity. The introduction of a visual loop detection module further optimizes the pose estimation of the LiDAR odometer and mapping algorithm, leveraging geometric and intensity information for precise pose determination.

## 4. Experiment

**Figure 6:**
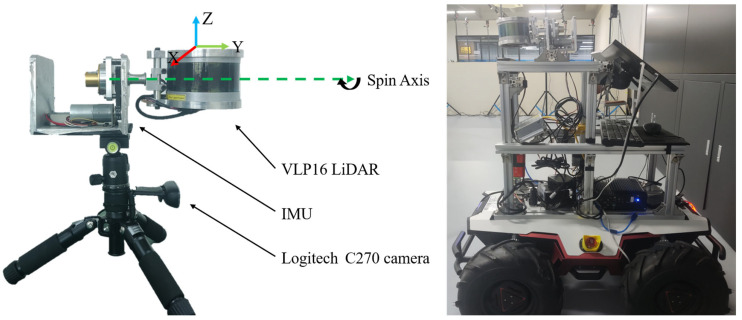
A rotary-driven LiDAR scanning system, which consists of a slip-ring mechanism with a stepper motor, enabling continuous forward scanning. An IMU and a camera are firmly attached to the platform, which are used for SLAM processing.

Since there are no publicly available datasets containing rotationally actuated LiDAR, IMU and camera data, we used the KITTI dataset to evaluate the performance of the algorithm LV-SLAM for the rotational lidar system. And we used the data collected in indoor and outdoor environments using the equipment shown in Figure 6. To evaluate the performance of the algorithm LV-SLAM for a rotary-driven LiDAR system, in the experiments, the sampling frequencies of the Velodyne-VLP-16 LIDAR (Velodyne Lidar, San Jose, CA, USA) and the ROS IMU (Wheeltec Co., Ltd., Dongguan, China) were 10 Hz and 200 Hz, respectively, and the angular speed of the stepper motor was set to 2.6. The computing device used was Intel^®^Core^TM^ i5-12400F (Santa Clara, CA, USA), and only the CPU was used in computing.

### 4.1. Dataset

The KITTI dataset has been widely used for the validation of vision, laser odometry and other algorithms. Three sequences from the KITTI dataset are selected for the evaluation of the proposed method: highway (Sequence #00), town (Sequence #05), and suburb (Sequence #07). The detailed information of these sequences is shown in Table 1, which is long enough to verify the SLAM algorithm and have multiple loops to evaluate the loop closure detection performance.

#### 4.1.1. Metric

When exploring the performance of SLAM algorithms, they are usually considered in terms of multiple dimensions such as complexity, runtime, and accuracy. Among these evaluation metrics, accuracy is undoubtedly the core concern. In the field of accuracy evaluation, absolute trajectory error (ATE) and relative position error (RPE) are the most widely used metrics.

The absolute trajectory error (ATE) directly calculates the difference between the true value of the system’s position and the estimated value of the SLAM system.

The true value is first aligned with the estimated value based on the timestamp of the pose, and then the difference between each pair of poses is computed and finally outputted in the form of a graph. ATE is the direct difference between the estimated pose and the true position, which provides a very intuitive response to the algorithmic accuracy and the global consistency of the trajectory.

Relative position error (RPE) is used to calculate the difference in the amount of change in position for the same two timestamps. After alignment with timestamps, both the true and estimated position are calculated for the amount of change in position at equal intervals, and then the difference in the amount of change is made to obtain the relative position error, and the change criterion is used to estimate the amount of drift in the system.

#### 4.1.2. Analysis of Experimental Results

**Figure 7:**
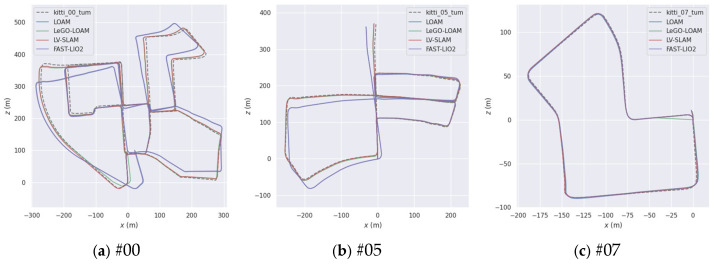
Motion trajectory positioning curves.

The comparison of the resulting trajectories from our method and three state-of-the-art algorithms—LOAM, LeGO-LOAM, and FAST-LIO2—is shown in Figure 7. It is evident that our method consistently produces trajectories that are closer to the ground truth across all sequences (#00, #05, #07), indicating reduced end-to-end drift errors. In particular, LOAM demonstrates significant global trajectory deviations, whereas our method maintains higher consistency with the true trajectory.

To quantify these differences, the absolute trajectory error (ATE) is computed for each method, as presented in Table 2. On average, our method achieves an end-to-end drift error of 2.47%, which is 6.01% lower than LOAM, 0.98% lower than FAST-LIO2, and 0.56% lower than LeGO-LOAM. For sequence #00, the end-to-end error is 1.9976, which represents a reduction of 4.91 over LOAM. Similar improvements are observed for sequences #05 and #07, as detailed in Table 2.

**Figure 8:**
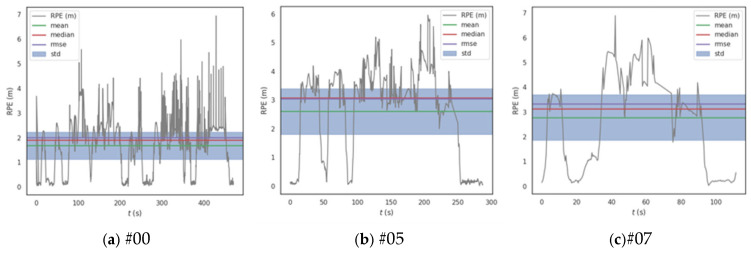
Relative trajectory error graph.

In addition to absolute positioning accuracy, the relative position error (RPE) is also evaluated to assess the short-term drift and local consistency. Table 3 summarizes the RPE metrics across the three sequences. As shown, our method consistently outperforms LOAM, LeGO-LOAM, and FAST-LIO2 in both mean and RMSEs. The average end-to-end relative drift error of our method is 1.27%, which is 2.10% lower than LOAM, 0.65% lower than FAST-LIO2, and 0.48% lower than LeGO-LOAM. The RPE translation results for each sequence are visualized in Figure 8.

### 4.2. Real-Time Demonstration on a Mobile Platform

**Figure 9:**
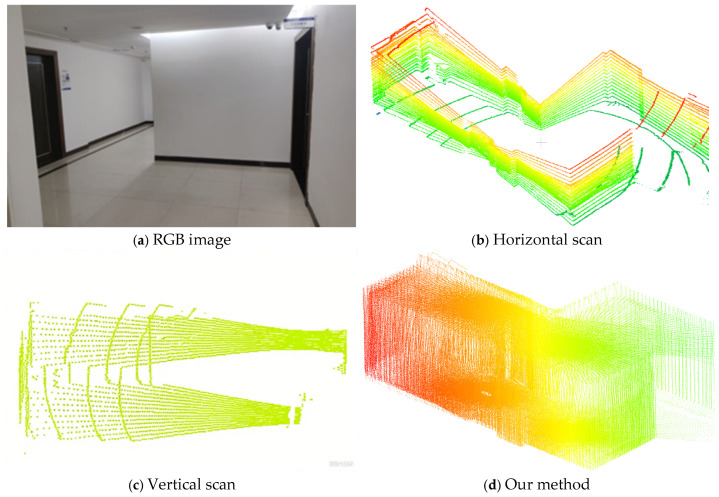
Figure (**a**) shows the RGB image, while (**b**–**d**) represent three different acquisition methods. Our method can obtain a more comprehensive 3D reconstruction.

The self-collected dataset includes indoor (laboratories and corridors) and outdoor (parking lot) environments. LiDAR data were captured using a calibrated Velodyne VLP-16 mounted on a rotating platform. The IMU (Wheeltec Co., Ltd., Dongguan, China) and Intel RealSense D435i RGB camera (Intel Corporation, Santa Clara, CA, USA) were hardware-synchronized using timestamp alignment with ROS middleware. All sensors were rigorously calibrated for extrinsic parameters. The calibration accuracy was validated via checkerboard tests for the camera and point-to-plane residuals for LiDAR-IMU alignment. The data were logged at stable temperatures, and noise filtering was applied post-recording. We performed indoor 3D reconstruction using LIDAR data captured at fixed points through horizontal, vertical, and rotational scanning. As shown in Figure 9, horizontal scanning mainly captured vertical walls but missed corridor roofs and floors. Vertical scanning focused on roofs and floors but lacked wall details. However, rotational scanning provided more complete coverage with fewer blind spots, resulting in better reconstruction quality. Experiments showed that rotational scanning excelled in maintaining the integrity of 3D reconstructions compared to horizontal and vertical scanning.

**Figure 10:**
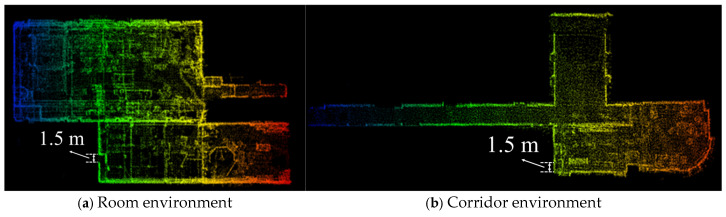
Performance testing of rotary-LIDAR system in indoor environments. The colors represent the changes in the y-axis position, with each color corresponding to a different range or value.

During both indoor and outdoor testing sessions, we employ a handheld rotary-driven LiDAR mapping system that enables us to swiftly construct accurate real-time 3D models of three distinctly different environments. Initially, we capture Data 1, which comprises two interconnected laboratory rooms, each with its unique set of features and challenges. Subsequently, we move on to Data 2, a lengthy and narrow corridor that poses significant positioning difficulties due to its sparse features and limited visual cues. Finally, we gather Data 3 from an outdoor parking lot, which presents an altogether different set of challenges related to natural lighting and open-air conditions. Our algorithm (LV-SLAM) exhibits excellent robustness even in texture degraded regions (Data 1 and Data 2). The generated 3D maps have a surprisingly high degree of visual consistency, capturing complex details with great accuracy. Our algorithm (LV-SLAM) exhibits excellent robustness even in texture degraded regions (Data 1 and 2). The generated 3D maps have a surprisingly high degree of visual consistency, capturing complex details with great accuracy, as shown in Figure 10.

**Figure 11:**
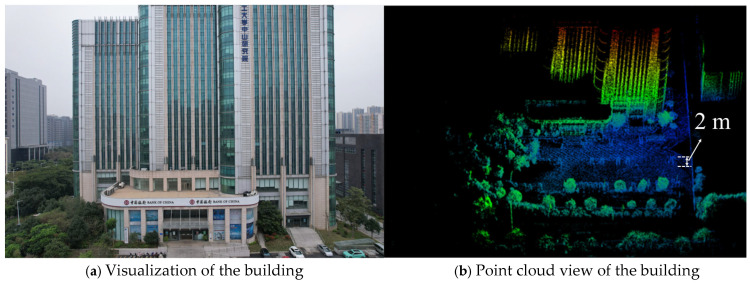
Aerial view of the Zhongshan Research Institute, Changchun University of Technology. Data were collected using a handheld system integrated with Jetson Nano and a portable outdoor mobile power supply, lasting 202 s and covering a ~120 m trajectory. The constructed map exhibits high visual consistency, with no noticeable blurring in structural details like walls, tree trunks, and vehicles.

Notably, the system also shows little visual degradation in areas that are blurred for long periods of time, such as walls, tree trunks, and vehicle surfaces, for Data 3, as shown in Figure 11. This proves the superior performance of our LiDAR mapping system, which is capable of quickly and accurately completing 3D point cloud map construction in a variety of environments.

To further evaluate the performance of our LV-SLAM system in challenging environments, we conducted a comparative mapping experiment in an underground parking lot. This environment features low illumination, repetitive structures, and sparse visual features—conditions that are typically unfavorable for visual or loosely-coupled SLAM systems.

**Figure 12:**
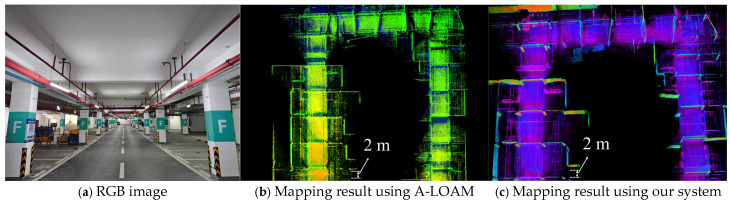
Mapping performance in an underground parking lot.

As shown in Figure 12a, the baseline A-LOAM system produced a distorted and incomplete map due to the lack of vertical LiDAR coverage and loop closure under large rotational drift. In contrast, our proposed rotary-driven LiDAR system combined with the LV-SLAM framework achieved significantly better mapping results (Figure 12b). The structural elements of the garage, such as walls, columns, and parked vehicles, are clearly preserved. Figure 12c shows the actual scene of the underground environment, confirming the complexity of the mapping challenge. The result demonstrates that our system maintains strong robustness and spatial completeness in low-light, texture-degraded environments, validating its adaptability for real-world deployment in semi-enclosed and GNSS-denied spaces.

In this study, the geometric accuracy of the reconstructed model is assessed by utilizing the height and width of key structural components, including doors, windows, and columns, within the buildings. The true dimensions of the structures are determined through manual measurements, with the average of multiple measurements serving as the ground truth. Correspondingly, the dimensions in the point cloud model are measured three times, and their average is taken as the observed value. Subsequently, the root mean square errors (RMSEs) in both the height and width directions of the reconstructed model are computed, as presented in Table 4.

Based on the experimental error analysis, it can be observed that the point cloud model generated by the algorithm proposed in this paper has an error within 5 cm compared to the actual building dimensions, indicating high accuracy. The main sources of error formation are threefold: first, the inherent measurement errors of the LiDAR itself; second, the positioning errors in the SLAM system can lead to minor deviations in the point cloud data generated during mapping; and third, the selection of different measurement points during the measurement process may introduce a certain degree of random error to the point cloud model accuracy. Overall, the method presented in this paper is capable of reconstructing building structures without compromising accuracy, thereby providing an efficient and reliable guarantee for high-precision 3D modeling.

## 5. Conclusions

In this work, we proposed a novel rotary-driven LiDAR system for achieving seamless 360° field-of-view scanning, coupled with a multi-sensor fusion SLAM framework (LV-SLAM) to enable accurate and robust 3D environment reconstruction. The system integrates a two-stage loop closure mechanism that leverages both visual and LiDAR-based features to enhance global trajectory consistency. Experimental validation on the KITTI dataset demonstrates that our method achieves a 2.05% improvement in ATE compared to LOAM, while maintaining lower RPE across sequences. Deployed on a handheld platform, the system produces real-time, visually consistent 3D maps in both indoor and outdoor settings.

Furthermore, geometric evaluation shows that reconstructed structural elements—such as doors, windows, and columns—match real-world dimensions with RMSE within 5 cm, confirming the high accuracy of the model. Future work will explore the integration of a joint visual–LiDAR BoW2-based loop closure module, deployment on mobile robotic platforms, and the incorporation of photoelectric encoders to measure rotational speed in real time. These enhancements aim to eliminate the assumption of uniform rotation and further improve system adaptability and robustness in dynamic or GNSS-denied environments.

## Figures and Tables

**Table 1:** KITTI dataset information.

Sequences	Parameters			
	Number of Frames	Length of Time (s)	Distance (m)	Maximum Speed (m s^−1^)
#00	4544	471	3682	12.9
#05	2762	288	2205	11.1
#07	1101	114	694	11.9

**Table 2:** Absolute trajectory error (ATE) of the methods with respect to the true values (all values are reported in meters).

Seq	Algorithm	Max	Mean	Median	Min	Rmse	Std
00	LOAM	19.678842	6.903108	5.442810	0.534717	8.480904	4.926747
	LeGO-LOAM	11.521786	2.449518	2.854562	0.067515	3.425754	1.802647
	FAST-LIO2	12.142564	4.215111	3.167574	0.029817	4.518875	2.475974
	OUR	10.530946	1.997565	2.076327	0.034538	2.476619	1.464027
05	LOAM	10.798714	2.734301	2.231552	0.804164	3.225685	1.709812
	LeGO-LOAM	6.149594	1.788600	1.986429	0.009964	2.112814	1.124676
	FAST-LIO2	5.037337	2.646398	2.614963	0.440284	2.774483	0.833268
	OUR	4.911035	1.160277	1.115179	0.001091	1.464198	0.893104
07	LOAM	16.691376	2.894979	1.209624	0.059581	4.625819	3.607949
	LeGO-LOAM	4.190354	2.413983	2.490165	0.193651	2.637720	1.063133
	FAST-LIO2	5.03964	2.589459	2.295412	0.069451	3.745251	1.180617
	OUR	4.231020	1.711174	1.647058	0.039654	2.061742	1.150071

**Table 3:** Relative position error (RPE) of the methods with respect to the true values.

Seq	Algorithm	Max	Mean	Median	Min	Rmse	Std
00	LOAM	7.572816	2.861219	3.293702	0.052028	3.371129	1.782677
	LeGO-LOAM	4.492446	1.024355	1.004043	0.004296	1.272371	0.754736
	FAST-LIO2	4.681298	1.892541	1.167574	0.009817	1.518875	0.475974
	OUR	3.872154	0.896217	0.923651	0.003125	1.058247	0.682519
05	LOAM	5.962009	2.606564	3.083750	0.012628	3.052875	1.589298
	LeGO-LOAM	3.267100	0.914836	1.012487	0.001205	1.110065	0.628745
	FAST-LIO2	2.192275	1.122483	1.195423	0.000652	1.198430	0.419840
	OUR	1.326789	0.587324	0.596213	0.125741	0.652189	0.172365
07	LOAM	6.893125	2.783081	3.128018	0.036888	3.337638	1.842359
	LeGO-LOAM	1.182524	0.658272	0.638349	0.149834	0.683565	0.184231
	FAST-LIO2	2.182669	0.901509	0.971551	0.001638	1.011673	0.459089
	OUR	1.326789	0.587324	0.596213	0.125741	0.652189	0.172365

**Table 4:** Accuracy error of the reconstructed model.

Model	Model Height	Model Width	Measured Height	Measured Width	Height Error	Width Error
Supporting Column	4.24 m	1.11 m	4.22 m	1.14 m	0.02 m	0.03 m
Window 1	2.50 m	1.20 m	2.55 m	1.23 m	0.05 m	0.03 m
Window 2	0.92 m	1.18 m	1.17 m	0.89 m	0.01 m	0.03 m
Door	2.13 m	2.05 m	2.17 m	2.00 m	0.04 m	0.05 m

## Data Availability

The original contributions presented in this study are included in the article. Further inquiries can be directed to the corresponding author.

## References

[1] Fan Y.C., Zheng L.J., Liu Y.C. (2018). 3D environment measurement and reconstruction based on LiDAR. Proceedings of the 2018 IEEE International Instrumentation and Measurement Technology Conference (I2MTC).

[2] Cadena C., Carlone L., Carrillo H., Latif Y., Scaramuzza D., Neira J., Reid I., Leonard J.J. (2016). Past, present, and future of simultaneous localization and mapping:Toward the robust-perception age. IEEE Trans. Robot..

[3] Liu X., Yuan C., Zhang F. (2022). Targetless extrinsic calibration of multiple small fov lidars and cameras using adaptive voxelization. IEEE Trans. Instrum. Meas..

[4] Raj T., Hanim Hashim F., Baseri Huddin A., Ibrahim M.F., Hussain A. (2020). A survey on lidar scanning mechanisms. Electronics.

[5] Lin J., Zhang F. (2021). R3live: A robust, real-time, rgb-colored, lidar-inertial-visualtightly-coupled state estimation and mapping package. arXiv.

[6] Liu J., Zheng J., Jia X., Li T., Zhang W. (2023). A tightly-coupled method of lidar-inertial based on complementary filtering. Meas. Sci. Technol..

[7] Kim G., Kim A. (2018). Scan context: Egocentric spatial descriptor for place recognition within 3d point cloud map. Proceedings of the 2018 IEEE/RSJ International Conference on Intelligent Robots and Systems (IROS).

[8] Yuanpei W., Xiaohong T. (2018). Visual slam loop closure detection based on improved orb. China Sci..

[9] Zhang G., Yan X., Ye Y. (2019). Loop closure detection via maximization of mutual information. IEEE Access.

[10] Harchowdhury A., Kleeman L., Vachhani L. (2018). Coordinated nodding of a 2d lida for dense 3d range measurements. IEEE Robot. Autom. Lett..

[11] Li Y., Hu Z., Li Z., Cai Y., Sun S., Zhou J. (2019). A single-shot pose estimation approach for a 2d laser rangefinder. Meas. Sci. Technol..

[12] Kaul L., Zlot R., Bosse M. (2016). Continuous-time three-dimensional mapping for micro aerial vehicles with a passively actuated rotating laser scanner. J. Field Robot..

[13] Bosse M., Zlot R., Flick P. (2012). Zebedee: Design of a spring-mounted 3-d range sensor with application to mobile mapping. IEEE Trans. Robot..

[14] Karimi M., Oelsch M., Stengel O., Babaians E., Steinbach E. (2021). Lola-slam: Lowlatency lidar slam using continuous scan slicing. IEEE Robot. Autom. Lett..

[15] Zhen W., Hu Y., Liu J., Scherer S. (2019). A joint optimization approach of lidar-camera fusion for accurate dense 3-d reconstructions. IEEE Robot. Autom. Lett..

[16] Yan L., Dai J., Zhao Y., Chen C. (2023). Real-time 3d mapping in complex environments using a spinning actuated lidar system. Remote Sens..

[17] Zhang J., Singh S. (2014). Loam: Lidar odometry and mapping in real-time. Robot. Sci. Syst..

[18] Shan T., Englot B. (2018). Lego-loam: Lightweight and ground-optimized lidar odometry and mapping on variable terrain. Proceedings of the 2018 IEEE/RSJ International Conference on Intelligent Robots and Systems (IROS).

[19] Shan T., Englot B., Meyers D., Wang W., Ratti C., Rus D. (2020). Lio-sam: Tightly coupled lidar inertial odometry via smoothing and mapping. Proceedings of the 2020 IEEE/RSJ International Conference on Intelligent Robots and Systems (IROS).

[20] Shan T., Englot B., Ratti C., Rus D. (2021). Lvi-sam: Tightly-coupled lidar-visual-inertial odometry via smoothing and mapping. Proceedings of the 2021 IEEE International Conference on Robotics and Automation (ICRA).

[21] Deng J., Wu Q., Chen X., Xia S., Sun Z., Liu G., Yu W., Pei L. (2023). Nerf-loam: Neural implicit representation for large-scale incremental lidar odometry and mapping. Proceedings of the IEEE/CVF International Conference on Computer Vision.

[22] Xing Z., Meng Z., Zheng G., Ma G., Yang L., Guo X., Tan L., Jiang Y., Wu H. (2025). Intelligent rehabilitation in an aging population: Empowering human-machine interaction for hand function rehabilitation through 3D deep learning and point cloud. Front. Comput. Neurosci..

